# Performance analysis of the BDSBAS-B1C message in trial operation stage

**DOI:** 10.1038/s41598-023-32846-4

**Published:** 2023-04-13

**Authors:** Jie Xin, Rui Guo, Jinping Chen, Yijun Tian, Yueling Cao, Yuchen Liu, Xiaojie Li, Dongxia Wang, Hongliang Cai

**Affiliations:** 1 Beijing Satellite Navigation Center, Beijing, 100094 China; 2 Shanghai Astronomical Observatory, Chinese Academy of Sciences, Shanghai, 200030 China; 3 Beijing Institute of Tracking and Telecommunication Technology, Beijing, 100094 China

**Keywords:** Space physics, Engineering

## Abstract

BeiDou Satellite-based Augmentation System (BDSBAS) has come into the trial operation stage since July, 2020. To evaluate the characteristic of the augmentation message in BDSBAS-B1C signal, the effectiveness of the message content was firstly analyzed, and then the validity of the broadcasting strategy was estimated. Finally, the accuracy of the user equivalent ranging error (UERE) and the single frequency positioning error with different correction parameters in BDSBAS-B1C message was evaluated. Based on the above analysis, the effectiveness of the augmentation message was preliminarily verified with the results showing that: (1) the BDSBAS-B1C message type, information content and update interval have basically met the international standard; (2) the accuracy of the UERE obtained with the augmentation message had an obvious improvement in contrast to that of the UERE obtained with the usual navigation message of the GPS satellites, and the ionospheric delay was one of the important factors which affected the accuracy of the UERE; (3) the positioning accuracy obtained with the augmentation message was also improved, and the improvement was more obvious in the service areas with high availability of the ionospheric parameters.

## Introduction

BeiDou navigation satellite system (BDS) has gone through the construction of BDS-1 and BDS-2^1^, and basically completed the system construction of the BDS-3 in July, 2020. It can provide seven kinds of service: basic positioning, navigation, and timing (PNT) service, global short message communication (GSMC) service, international search and rescue (SAR) service, satellite-based augmentation service, ground augmentation service, precise point positioning (PPP) service and regional short-message communication (RSMC) service. Up to now, the satellite-based augmentation service of the BDS-3 is still in trial operation stage.

Since 2010, BDS-2 has been providing augmentation message for users in China and the surrounding areas via the BDS-2 geostationary earth orbit (GEO) satellites. The message includes the equivalent clock correction, grid ionospheric correction and the corresponding integrity parameters. As the format of the BDS-2 is designed as page, it is broadcasted along with the basic navigation message. Therefore, BDS-2 is a system which can provide the basic navigation and satellite-based augmentation service simultaneously. It can significantly improve the service precision of the specific area. In recent years, the supplement parameters, such as the comprehensive partition correction parameters and the orbit correction parameters, are added in the message of the BDS-2 to further improve the service performance. Whereas, the equivalent clock correction parameters contain both the error of satellite clock and orbit and the ionospheric grid is a kind of serried grid. All of the parameters are broadcasted in the B1I, B2I or B3I signal. The constraints of the signal features and message format make it hard to meet the certification requirements of the international civil aviation.

As the new generation of the BDS, the BDS-3 has stepped further in the integration of the platform, data, technology and terminal, and paid more attention in compatibility and interoperability with other international systems^2^. It designs a new message structure and broadcasted different service message in different signals, in accordance with the International Civil Aviation Organization (ICAO) standards.

Currently, the SBASs in service are the USA’s Wide Area Augmentation System (WAAS), EU’s European Geostationary Navigation Overlay System (EGNOS), Japanese Michibiki Satellite-based Augmentation System (MASAS), and Indian GPS-aided GEO augmented navigation system, which are all SF SBASs^3^. Owing to the effect of the ionospheric anomalies, it’s difficult to satisfy the requirements of Category I precision approach (CAT-I). Only WAAS and EGNOS have satisfied the requirements of the localizer performance with vertical guidance at 200 feet decision height^4^^,^^5^. Whether the BDSBAS could gain the identification of the ICAO, extensive testing is required to assess the various indicators, such as PE, alarm time and integrity threat.

As the launching of the last BDS-3 GEO satellite in June, 2020, the BDSBAS-B1C messages has been broadcasted steadily. BDSBAS formally steps into the trial operation stage since July, 2020. By the end of 2022, the BDSBAS is still in the testing stage. To preliminarily analyze the performance of the BDSBAS-B1C message, we researched on the content of the broadcasted message, deduced the broadcasting strategy and evaluated the accuracy of the user equivalent ranging error (UERE) and the SF positioning error (PE) with the support of different correction parameters in BDSBAS-B1C.

## BDSBAS overview

**Figure 1:**
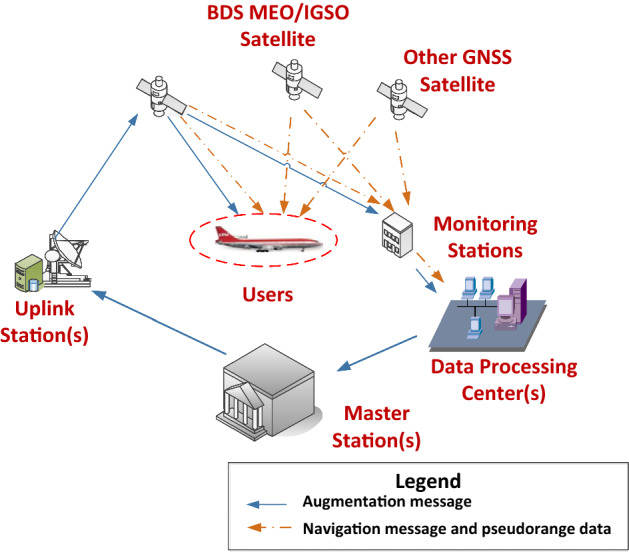
Illustration of the BDSBAS architecture. It consists of three parts: a space segment, which includes GEO satellites and other global navigation satellite system (GNSS) satellites; a ground segment, which includes monitoring stations, data processing centers, master stations and uplink stations; a user segment, which mainly includes the civil aviation users.

BDSBAS is an important part of the BDS-3, mainly applied in civil aviation, maritime and railway. As shown in Fig. 1, the BDSBAS mainly consists of three parts: the space segment, the ground segment and the user segment.

### Space segment of the BDSBAS

**Figure 2:**
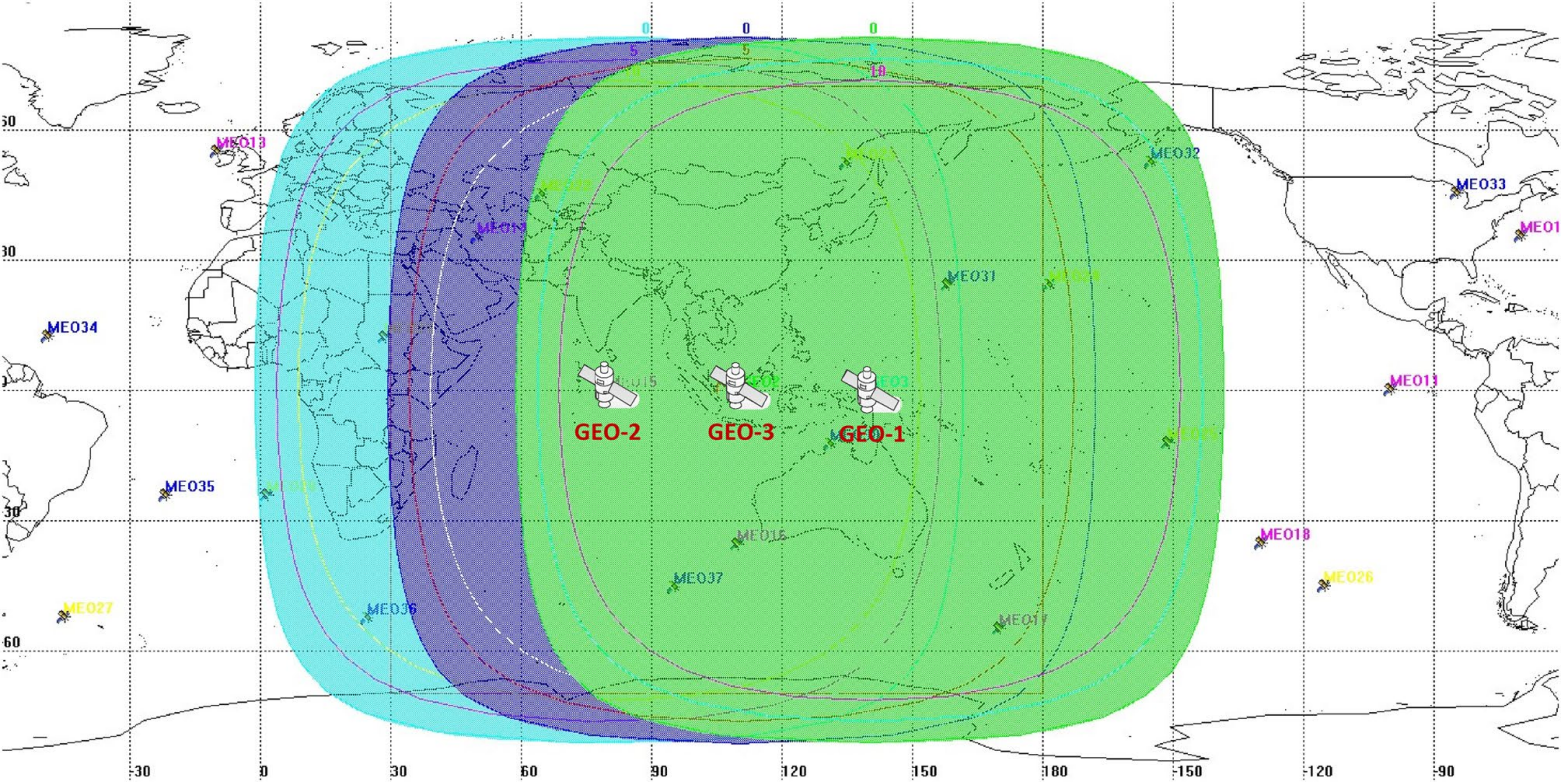
Illustration of the coverage areas for the BDS-3 GEO satellites. The blue, purple and green area shown in the figure are the service areas of the GEO satellites, which are located at 80 °E, 110.5 °E and 140 °E, respectively.

The space segment consists of three BDS GEO satellites. As showed in Fig. 2, these satellites operate in orbit at an altitude of 35,786 km and are located at 80 °E, 110.5 °E, and 140 °E, which are using Pseudo Random Noise (PRN) code 144, 143 and 130, respectively.

The augmented satellites will include BDS-3 medium orbit earth (MEO), inclined geosynchronous orbit (IGSO) satellites and other GNSS satellites, such as global position system (GPS), Galileo satellite navigation system (Galileo) and global navigation satellite system (GLONASS) satellites. At present, the augmentation message of the GPS satellites is broadcasted in BDSBAS-B1C signal and BDSBAS-B2a signal, while the augmentation message for BDS-3 satellites is only broadcasted in BDSBAS-B2a signal.

### Ground segment of the BDSBAS

The ground segment of the BDSBAS is constructed with the BDS-3 ground segment, including monitoring stations, data processing centers, main control stations and injection stations. The distribution of the stations has not been published.

The space–time reference system maintained by the BDSBAS SF service keeps consistent with WAAS. The coordinate system of BDSBAS is WGS-84. The deviation of BDSBAS SF service network time (SNT) to GPS Time (GPST) is within 50 ns.

### User segment of the BDSBAS

The civil terminal of the BDSBAS mainly includes the airborne terminal and land terminal. The airborne terminals can be applied in commercial aviation, general aviation and unmanned aerial vehicles. The land-based terminals can be used for the low dynamic land-based or maritime equipment.

## Broadcasting status of the BDSBAS-B1C message

The current SBASs are independent and regional operating systems, which mainly provide augmentation service to civil aviation users within the coverage area of their respective systems^3^. How to realize the seamless connection in the overlapping area has become an inevitable issue for the performance improvement of the SBAS services^6^. The consistency of the signal system and message structure is the basic requirement.

The realization of the BDSBAS-B1C message is depend on the BDS signal-in-space interface control document: satellite based augmentation system service signal BDSBAS-B1C, which strictly follows the Satellite Based Augmentation System (SBAS) Standards and Recommended Practices (SARPs) of ICAO “Convention on International Civil Aviation” Annex 10 Aeronautical Telecommunications Volume I Radio Navigation Aids^7^.

Based on the signal characteristics and message structure described in the BDSBAS-B1C document, the message content and broadcasted strategy can be analyzed with the actual message.

### Message content of the BDSBAS-B1C

The broadcasted BDSBAS-B1C message mainly includes fast corrections, long-term satellite error corrections, ionospheric corrections and the corresponding integrity parameters. Based on the message broadcasted from Dec 11 to 31, 2020, Table 1 gives the broadcasted message type (MT), information definitions, and content descriptions. The MT0 information was broadcast at least once every 6 s to indicate that the system was still in the testing stage. The other MT represented different kinds of broadcasted message, for example, MT1 symbolizes the message of PRN mask assignment.

**Table 1:** The message types and content broadcasted in the BDSBAS-B1C signal.

Message types	Definition	Content descriptions
MT0	Do not use for safety application	It identified that the BDSBAS was under testing
MT1	PRN mask assignment	It contained the PRN masks of 32 GPS satellites
MT2–MT4	Fast corrections	It included fast corrections and user differential range error indicator (UDREI). Fast corrections provided pseudo-range corrections include the equivalent ranging effect caused by satellite position and clock error. The UDREI indicated the accuracy of combined fast and long-term error corrections, not included the accuracy of the ionospheric delay correction
MT6	Integrity information	It included the several UDREIs of the abnormal satellites
MT7	Fast corrections degradation factor	It was temporarily broadcasted as fixed value (the default value was 15). It indicated the maximum fast correction update interval or user time-out interval
MT9	GEO navigation message	It contained the satellite ephemeris of the PRN130, PRN143 or PRN144
MT10	Degradation factor	It was temporarily broadcasted as fixed values
MT17	GEO satellite almanacs	It contained the satellite almanac of the PRN130, PRN143 and PRN144
MT18	Ionospheric grid point masks	It contained the information of the ionospheric grid points (IGPs) in the band 6, 7 and 8 Each Type. Each packet contains one band information
MT25	Long term satellite error corrections	It contained the positioning and clock correction information of the satellites within the service area. Long term satellite error corrections provide the estimated errors for slow varying satellite ephemeris and clock errors with respect to WGS-84 ECEF coordinates. Each packet contains the information of four satellites
MT26	Ionospheric delay corrections	It temporarily contained a fixed number of IGPs and grid ionospheric vertical error indicator (GIVEI) (the number is 109). It provided the users with vertical delays (relative to an L1 signal) and their accuracy at geographically defined IGPs. Each packet contains the information of 15 ionospheric grids
MT28	Clock-ephemeris covariance matrix message	It contained the clock-ephemeris covariance information of the satellites within the service area. The covariance matrix was a function of satellite location, reference station observational geometry, and reference station measurement confidence. Each packet contains the information of two satellites
MT63	Null message	It was a filler message if no other message was available for broadcast for a one-second time slot

Not all messages defined in the BDSBAS-B1C message were broadcasted, such as the offset parameters of BDSBAS SNT and UTC (MT12) and the fast corrections/long term satellite error corrections (MT24). These messages were also not broadcasted regularly in WAAS. Whereas, EGNOS, a system which could augment GPS and GLONASS, provided not only the MT 12 but also the SBAS service message (MT 27). As the augmented object of the BDSBAS was also the GPS and GLONASS, the MT 12 and MT 27 may also be broadcasted in BDSBAS-B1C signal as EGNOS.

**Figure 3:**
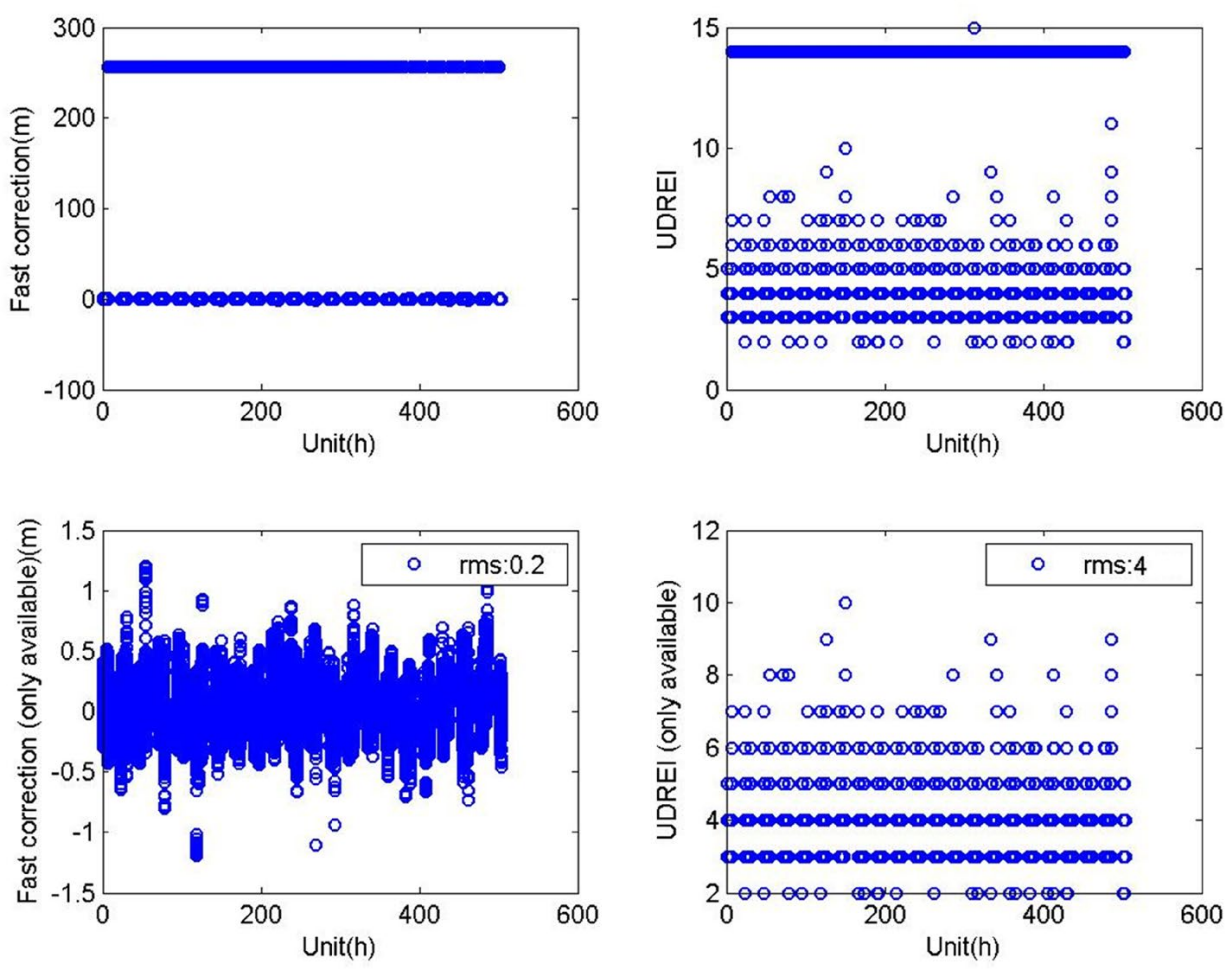
Fast correction and integrity information of the PRN20 satellite broadcasted from Dec 11 to 31, 2020 (top left: broadcasted fast correction parameters for 504 h; bottom left: available fat correction parameters; top right: broadcasted UDREI for 504 h; bottom right: available UDREI).

**Figure 4:**
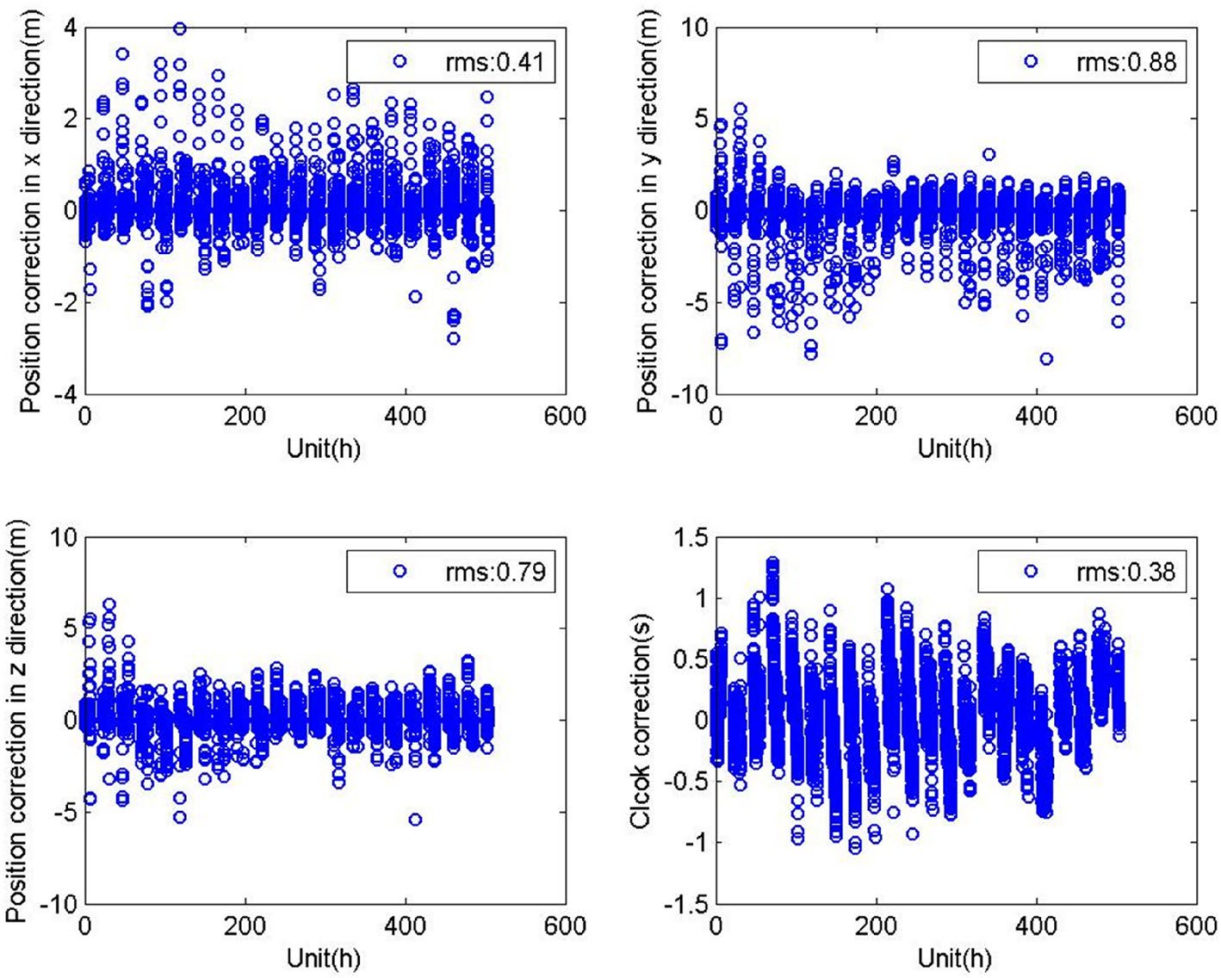
Positioning and clock correction information of the PRN20 satellite broadcasted from Dec 11 to 31, 2020 (top left: broadcasted position correction parameters in the x direction; bottom left: broadcasted position correction parameters in the z direction; top right: broadcasted position correction parameters in the y direction; bottom right: broadcasted clock correction parameters).

The details of the broadcasted messages are further described in the following paragraphs:

1. The correction parameters and the integrity parameters of the 32 GPS satellites have been broadcasted regularly. When the satellites were not in the BDSBAS service area or an alarm event occurs, the correction information of the corresponding satellites should not be broadcasted or set as a default value. Some alarm events have been monitored, which were mainly caused by the low availability as the satellite got close to the service boundary.
2. As shown in Table 2, it could be seen that when the satellites were within the BDSBAS service area, the statistic values of the orbit correction (three-dimensional), clock correction and fast correction parameters were about 1.6 m, 0.48 m and 0.15 m, respectively, and the corresponding UDREI was about 4, meaning that the maximum value of the user differential ranging error (UDRE) was 2.25 m. The broadcasted fast correction and integrity information of the PRN20 satellite was shown in Fig. 3 and the positioning and clock correction information from Dec 11 to 31, 2020 was shown in Fig. 4. Compared with the parameters broadcasted by the adjacent MASAS, the values were basically in the same magnitude.

**Table 2:** Fast correction, positioning and clock correction information of GPS satellites broadcasted from Dec 11 to 31, 2020.

PRN	Fast correction and integrity information (only available)		Position correction			Clock correction (s)
	Fast correction (m)	UDREI	X (m)	Y (m)	Z (m)	
1	0.17	4	0.67	1.28	0.87	0.41
2	0.16	4	0.34	1.13	0.94	0.43
3	0.22	4	0.73	1.43	1.00	0.43
4	0.14	4	0.63	1.20	0.92	0.30
5	0.20	4	0.38	1.40	1.32	0.36
6	0.21	4	0.34	1.21	0.96	0.50
7	0.17	4	0.47	0.99	0.62	0.64
8	0.39	4	0.56	1.22	0.98	0.75
9	0.17	4	0.79	1.63	1.16	0.36
10	0.18	4	0.36	0.74	0.63	0.60
11	0.23	4	0.58	1.61	1.06	0.51
12	0.17	4	0.72	1.48	0.82	0.37
13	0.16	4	0.57	1.56	0.87	0.57
14	0.21	4	0.54	0.72	0.89	0.92
15	0.15	4	0.66	1.28	1.12	0.29
16	0.17	4	0.59	1.48	1.33	0.33
17	0.26	4	0.39	1.02	1.06	0.64
18	0.13	4	0.43	0.78	0.59	0.47
19	0.15	4	0.56	0.87	0.77	0.38
20	0.15	4	0.41	0.88	0.63	0.31
21	0.19	4	0.88	1.28	1.01	0.40
22	0.18	4	0.75	1.48	0.98	0.33
23	0.17	4	0.53	0.68	0.70	0.53
24	0.36	4	0.61	1.51	0.88	0.99
25	0.18	4	0.64	1.53	1.11	0.41
26	0.16	4	0.53	0.98	0.82	0.37
27	0.17	4	0.70	1.78	0.96	0.38
28	0.22	4	0.44	0.97	0.70	0.65
29	0.28	4	0.86	1.65	1.09	0.41
30	0.18	4	0.65	1.27	0.98	0.47
31	0.19	4	0.66	0.84	0.87	0.39
32	0.21	4	0.71	0.77	0.81	0.42
average	0.20	4	0.57	1.21	0.92	0.48

**Figure 5:**
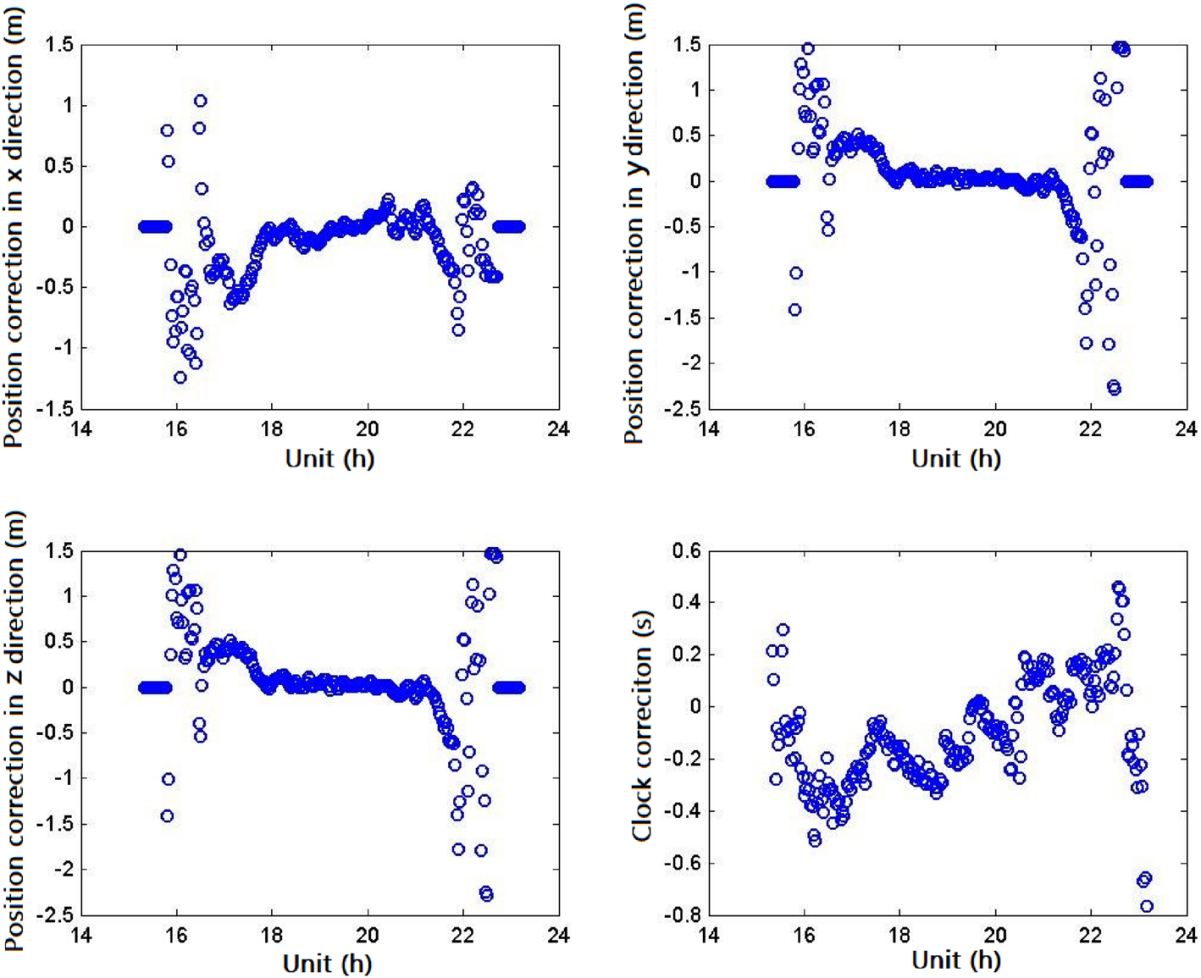
Positioning and clock correction information of the PRN20 satellite broadcasted on Dec 11. (top left: broadcasted position correction parameters in the x direction; bottom left: broadcasted position correction parameters in the z direction; top right: broadcasted position correction parameters in the y direction; bottom right: broadcasted clock correction parameters).

The positioning and clock correction information on Dec 11, 2020 was shown in Fig. 5. It could be seen that as the satellite came into/out of the service area, the position corrections in different directions were zero while the clock correction was not. It can be deduced that the resolving condition of the position corrections was stricter than the clock correction and the resolving of the orbit and clock error was separated.

**Figure 6:**
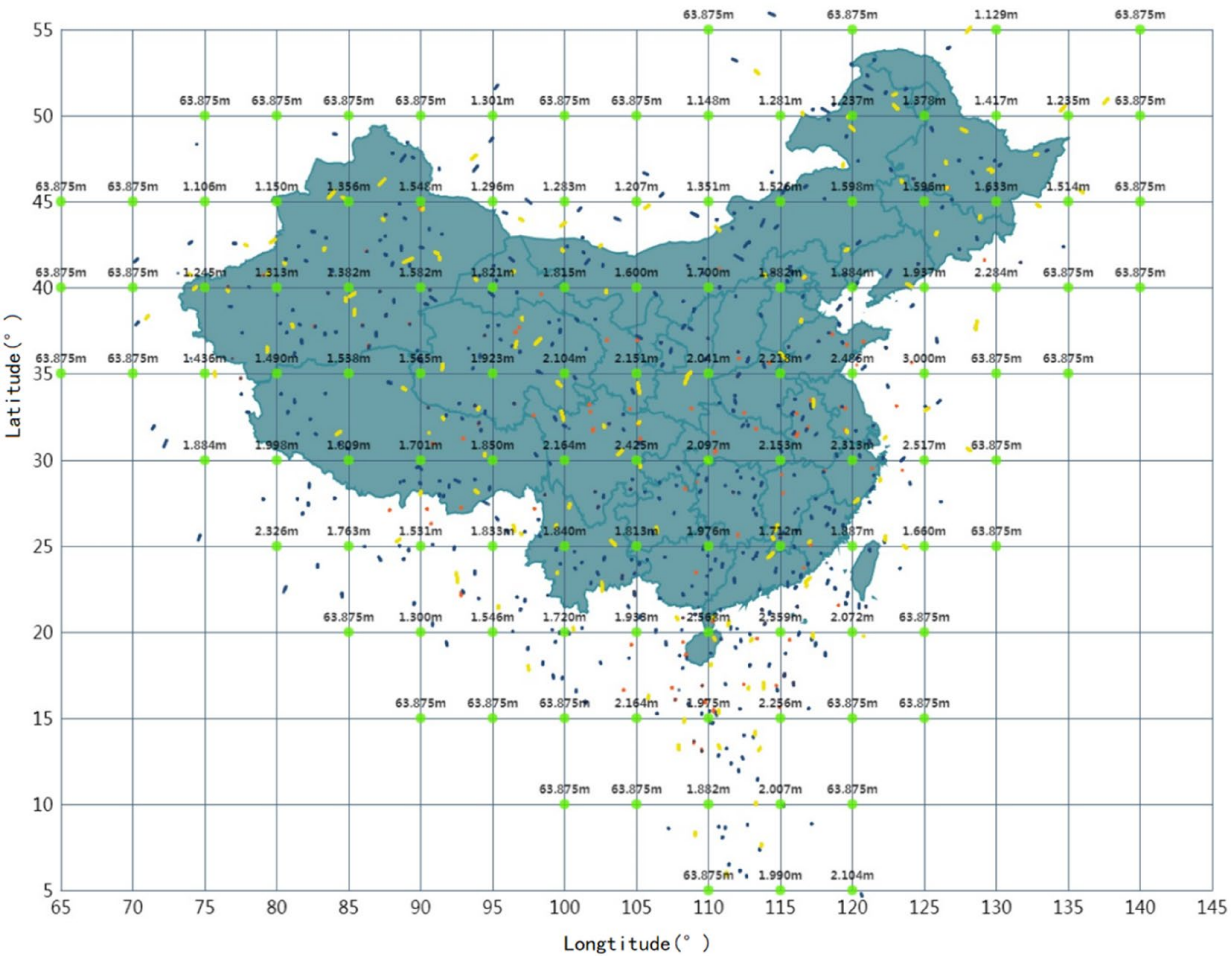
Ionospheric correction values of the fixed IGPs. The available value of the ionospheric correction parameters is about 2 m around the BDSBAS service area, and the default value of the ionospheric correction parameter is 63.875 m when it cannot be calculated.

(3) The ionospheric corrections of the fixed IGPs were shown in Fig. 6 which cannot be guaranteed with 100% availability temporarily, especially the service boundary areas. It can be deduced that the BDSBAS may also adopt the inverse distance weighting algorithm, which was initially adopted by the initial WAAS to calculate the ionospheric delay of the IGPs^8,9^ as the low availability of the boundary area. As the development of WAAS, it adopted the Kriging interpolation algorithm to calculate the ionospheric delay, which could greatly reduce the reliance on the monitoring stations and improve the IGPSs availability in the boundary areas^10^^,^^11^. While, the applicability of this algorithm for the BDSBAS should be further demonstrated.

Normally, the broadcasted ionospheric grids are fixed. To improve the service performance and ensure the availability of each grid, WAAS has made four major adjustments. Whereas, the availability of the ionospheric grids surrounding the BDSBAS service boundary were rather low. Therefore, BDSBAS may further adjust the ionospheric grids.

(4) MT28 contained an upper triangular $E$ and a scale factor ($SF_{1}$). The calculation method is shown in the following formula (1) and (2). Then, the observation covariance matrix ${\text{C}}$ which reflected the clock error $\delta t$ and the ephemeris errors in three directions ($\delta x,\,\delta y,\,\delta z$) could be obtained. The resolving process was shown in formula (3):
  1 $$ E = \left[ {\begin{array}{*{20}c} {E_{1,1} } & {\quad E_{1,2} } & {\quad E_{1,3} } & {\quad E_{1,4} } \\ 0 & {\quad E_{2,2} } & {\quad E_{2,3} } & {\quad E_{2,4} } \\ 0 & {\quad 0} & {\quad E_{3,3} } & {\quad E_{3,4} } \\ 0 & {\quad 0} & {\quad 0} & {\quad E_{4,4} } \\ \end{array} } \right] $$
  2 $$ R = E \cdot SF_{1} $$
  3 $$ C = R^{{\text{T}}} R $$

In the formula, $E_{{}}$ referred to the clock-ephemeris covariance matrix and $E_{1,1}$ to $E_{1,4}$ referred to the matrix elements; $SF_{1}$ was a scale factor whose value was 2^(scale exponent-5)^; $C$ referred to a relative covariance matrix of $R$.

Therefore, $E_{1,4}$, $E_{2,4}$ and $E_{3,4}$ broadcasted in MT28 are the elements concerned with $(\delta x,\delta t),(\delta y,\delta t),(\delta z,\delta t)$ respectively. As the values of the $E_{1,4}$, $E_{2,4}$ and $E_{3,4}$ parameters are all 0, it could be further deduced that the BDSBAS adopted a method with the resolving of the orbit and clock error separated. It was a valid method to reduce the resolving dimension.

(5) The broadcasted degradation factors were all constant values in MT7 and MT10. The factors concerning the ionosphere took the same values with SDCM and other factors took the values of WAAS. The rationality of the value should be further verified.
(6) The MT9 and MT17 provided the satellite position, velocity, clock time and an accuracy exponent of the BDS GEO satellite. Considering that the BDS GEO satellites did not provide ranging service, the accuracy exponent was set as a fixed value of “15” which indicated that the satellite ranging signal should not be used.

### Broadcasted strategy of the BDSBAS-B1C

**Figure 7:**
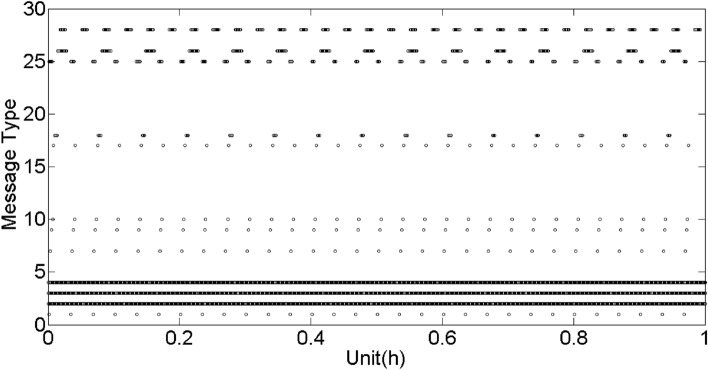
Update interval of the message types from MT1 to MT28 within one hour (Dec 11, 2020). Each type had a fixed broadcasting interval.

Up to now, BDSBAS has not published the broadcasted strategy of the BDSBAS-B1C message, so does WAAS and EGNOS. Though the RTCA DO-229E^12^ has constrains the maximum update interval of each type, each SBAS has adopted different broadcasting strategies. Compared with WASS and EGNOS, the update interval of BDSBAS-B1C message was rather fixed. The update interval of the BDSBAS augmentation message was shown in Table 3 and Fig. 7.

**Table 3:** Update interval of the augmentation message in BDSBAS.

Message types	Maximum update interval	Update interval in BDSBAS
MT0	6 s	At least once every 6 s
MT1	once every 120 s/updated	Once every 120 s
MT2–MT4	6 s	Once every 6 s
MT7	120 s	Once every 120 s
MT9	120 s	Once every 120 s
MT10	120 s	Once every 120 s
MT17	300 s	Once every 120 s
MT18	300 s/updated	Three times every 240 s, which concerns with the number of the broadcasted bands
MT25	120 s	Several times every 120 s, which concerns with the number of the visible satellites
MT26	300 s	Nine times every 240 s, which concerns with the number OF the broadcasted ionospheric grids
MT28	120 s	Several times every 120 s, which concerns with the number of the visible satellites
MT63	–	Random

Through the analysis of the message types broadcasted on Dec 11, 2020 (Fig. 5), it could deduced that the specific broadcasting strategy was as follows:

1. The update interval of the MT2–4 was 6 s, and the update cycle could be described as the formula (4).
  4 $$ \left\{ \begin{gathered} t_{{{\text{MT2}}}} = t_{0} [6] + 3 \hfill \\ t_{{{\text{MT3}}}} = t_{0} [6] + 4 \hfill \\ t_{{{\text{MT4}}}} = t_{0} [6] + 5 \hfill \\ \end{gathered} \right. $$

In the formula, $t_{{{\text{MT2}}}}$, $t_{{{\text{MT3}}}}$ and $t_{{{\text{MT4}}}}$ referred to the broadcasted time of the MT2–4; $t_{0} [6][6]$ referred to the time of day $t_{0}$ modulo 6 s.

(2) The update interval of the MT1, MT7, M9, M10 and M17 was 120 s, and the update cycle could be described as the formula (5).
  5 $$ \left\{ \begin{gathered} t_{{{\text{MT1}}}} = t_{0} [120] + 20 \hfill \\ t_{{{\text{MT7}}}} = t_{0} [120] + 26 \hfill \\ t_{{{\text{MT9}}}} = t_{0} [120] + 32 \hfill \\ t_{{{\text{MT10}}}} = t_{0} [120] + 38 \hfill \\ t_{{{\text{MT17}}}} = t_{0} [120] + 44 \hfill \\ \end{gathered} \right. $$

In the formula, $t_{{{\text{MT1}}}}$, $t_{{{\text{MT7}}}}$, $t_{{{\text{MT9}}}}$, $t_{{{\text{MT10}}}}$ and $t_{{{\text{MT17}}}}$ referred to the broadcasted time of the MT1, MT7, MT9, MT10 and MT17; $t_{0} [120]$ referred to the time of day $t_{0}$ modulo 120 s.

(3) The update interval of the MT18 and MT26 was 240 s, and the update cycle could be described as the formula (6).
  6 $$ \left\{ \begin{gathered} t_{{{\text{MT18}}\_part1}} = t_{0} [240] + 50 \hfill \\ t_{{{\text{MT18}}\_part2}} = t_{0} [240] + 56 \hfill \\ t_{{{\text{MT18}}\_part3}} = t_{0} [240] + 62 \hfill \\ \end{gathered} \right. $$

In the formula, $t_{{{\text{MT18}}\_part1}}$, $t_{{{\text{MT18}}\_part2}}$ and $t_{{{\text{MT18}}\_part3}}$ referred to the broadcasted time of the three groups of the MT18; $t_{0} [240]$ referred to the time of day $t_{0}$ modulo 240 s.

7 $$ \left\{ {\begin{array}{*{20}l} {t_{{{\text{MT26}}\_part1}} = t_{0} [240] + 20 + (1 + 7) \times 6} \hfill \\ {t_{{{\text{MT26}}\_part2}} { = }t_{0} [240] + 20 + (2 + 7) \times 6} \hfill \\ \cdots \hfill \\ {t_{{{\text{MT26}}\_part9}} = t_{0} [240] + 20 + (m + 7) \times 6} \hfill \\ \end{array} } \right. $$

In the formula, $t_{{{\text{MT}}26\_part1}}$, $t_{{{\text{MT}}26\_part2}}$… and $t_{{{\text{MT26}}\_part9}}$ referred to the broadcasted time of the nine groups of the MT26; $t_{0} [240]$ referred to the time of day $t_{0}$ modulo 240 s; $m$ referred to the broadcasted groups of the MT26.

(4) The update interval of the MT25 and M28 was 120 s, and the number of broadcast groups in each update cycle was relative to the visibility and availability of the satellites.
(5) When there was no valid information generated, the MT0 or MT63 would be broadcast.

## Evaluation of the message performance

As BDSBAS is still in testing stage, the service boundary has not been published. Assuming the service area of the BDSBAS SF service was within China, some testing terminals were set in the central and boundary areas of China to test the accuracy of the UERE and the SF PE with the different correction parameters in BDSBAS-B1C message.

### UERE evaluation with/without the support of the BDSBAS-B1C message

The pseudorange residual error was the difference between the raw pseudorange and the reference range. The reference range could be gain with the true range between the corrected satellite position and the receiver, all the broadcasted corrections, tropospheric delay, receiver clock bias, and multipath. Therefore, the UERE mainly comprised the error in the space segment and the pseudorange residual error in the ground segment. It could be calculated as the formula (8).

8 $$ UERE_{{{\text{SBAS}}}} = (\rho^{\prime} - \rho ) - (c \cdot \delta t_{r} - c \cdot \delta t_{s} ) - d_{tro} - d_{rel} - d_{mul} - (d_{orb} + d_{clk} + d_{fast} + d_{grid} ) - \varepsilon_{p} $$

In the formula, $\rho^{\prime}_{{}}$ refers to the observed pseudorange; $\rho$ refers to the geometrical distance between the receiver and the satellite. The points of the receiver should be measured by the professional surveying team and used for the precise orbit determination; $c$ refers to the velocity of light; $\delta t_{r}$ refers to the clock error of the receiver; $\delta t_{s}$ refers to the clock error of the satellite; $d_{tro}$ refers to the tropospheric delay; $d_{rel}$ refers to the delay caused by the relativistic effect; $d_{mul}$ refers to the multipath delay; $d_{orb}$ refers to the orbit error calculated by the orbit correction parameters; $d_{clk}$ refers to the clock error calculated by the clock correction parameters; $d_{fast}$ refers to the error calculated by the fast correction parameters; $d_{grid}$ refers to the error calculated by the ionospheric correction parameters of the IGPs; $\varepsilon_{p}$ refers to the residual error. We use the value of the 95% percentile UERE, a 95% quantile statistics, as the evaluation accuracy of the day.

As to UERE of the GPS standard positioning service (SPS), the evaluation equation can be described as the formula (9).

9 $$ UERE_{{{\text{SPS}}}} = (\rho^{\prime}_{{}} - \rho ) - (c \cdot \delta t_{r} - c \cdot \delta t_{s} ) - d_{tro} - d_{rel} - d_{mul} - d_{{{\text{ion}}}} - \varepsilon_{p} $$

In the formula, $d_{{{\text{ion}}}}$ refers to the error calculated by the ionospheric model parameters which are broadcasted in the navigation message.

It could be seen that the difference between the $UERE_{{{\text{SBAS}}}}$ and the $UERE_{{{\text{SPS}}}}$ was not only in the augmentation parameters used, but also in the correction mode of the ionospheric error.

**Figure 8:**
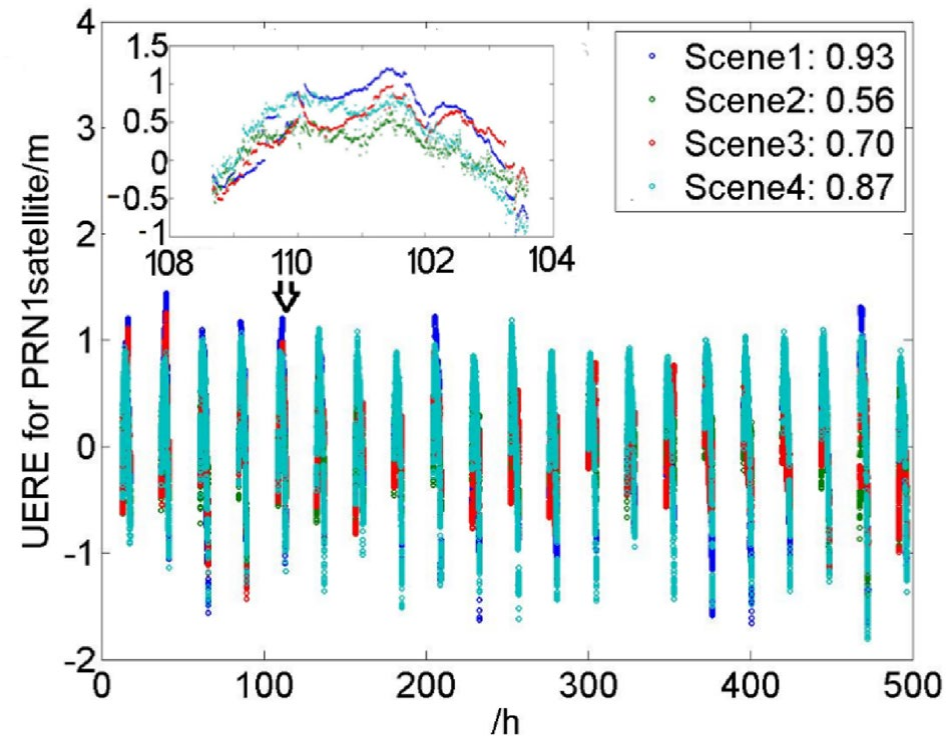
Evaluation of the UERE under different scenes for PRN1 satellite in Beijing from Dec 11 to 21, 2020.

To analyze the UERE of the GPS satellites in different correction modes, four groups of experiments were carried out with the observation data obtained by the known point in Beijing, Sanya, Chengdu, Shantou, Harbin and Urumchi from Dec 11 to 31, 2020. The four specific scenes were constructed as follows: (1) according to the Eq. (9), the observation data and the navigation message of the GPS satellites were used to evaluate the accuracy of the GPS SPS UERE; (2) according to the Eq. (8), the evaluation of the UERE was determined by using all correction parameters broadcasted in the BDSBAS-B1C; (3) on the basis of the Eq. (8), the evaluation of the UERE was determined by only using ionospheric delay correction parameters of the IGPs broadcasted in the BDSBAS-B1C; (4) on the basis of the Eq. (8), the evaluation of the UERE was determined by only using orbit and clock correction parameters broadcasted in the BDSBAS-B1C. The results were shown in Tables 4, 5 and Fig. 8.

**Table 4:** Evaluation of the UERE under different scenes (Beijing, Sanya and Chengdu, unit: m).

PRN	Beijing				Sanya				Chengdu			
	Scene1	Scene2	Scene3	Scene4	Scene1	Scene2	Scene3	Scene4	Scene1	Scene2	Scene3	Scene4
1	0.93	0.56	0.70	0.87	2.15	0.97	1.02	2.06	1.17	0.74	0.84	1.07
2	1.02	0.72	0.74	0.95	1.18	0.54	0.69	0.97	1.13	1.07	1.10	1.04
3	0.53	0.34	0.70	0.36	1.91	0.96	1.01	1.78	0.65	0.89	1.01	0.51
4	0.51	0.24	0.49	0.33	1.35	0.65	0.71	1.29	0.90	0.47	0.64	0.69
5	1.30	0.48	1.07	0.81	1.35	0.77	1.23	0.78	1.29	0.46	1.00	0.76
6	0.71	0.51	0.56	0.69	1.02	0.97	0.86	1.13	0.90	0.79	0.77	0.94
7	1.40	0.37	1.21	0.58	1.48	0.55	1.38	0.65	1.51	0.44	1.37	0.60
8	1.77	0.32	1.66	0.64	1.67	0.55	1.81	0.79	2.21	0.58	1.93	1.04
9	0.00	0.46	0.32	0.13	0.93	0.66	0.54	1.27	0.49	0.77	0.61	0.72
10	0.92	0.25	0.54	0.70	0.58	0.58	0.79	0.47	1.67	0.57	0.73	1.79
11	1.40	0.42	1.04	0.82	1.65	0.46	1.07	1.09	1.63	0.59	1.18	1.00
12	0.53	0.47	0.66	0.33	3.01	1.57	1.69	2.79	0.89	0.77	0.93	0.75
13	0.82	0.47	0.78	0.77	1.09	0.80	0.79	1.18	0.95	0.75	0.76	0.97
14	1.25	0.67	1.00	0.44	1.19	0.62	1.34	0.42	1.15	0.55	0.96	0.49
15	0.70	0.39	0.54	0.56	0.62	0.43	0.65	0.41	0.99	0.59	0.77	0.78
16	1.17	0.35	0.72	0.95	0.82	0.46	0.65	0.49	2.67	0.83	1.00	2.68
17	1.99	0.94	1.75	1.15	1.93	1.02	1.80	1.32	2.15	1.35	2.14	1.31
18	0.89	0.44	0.64	0.76	1.35	0.73	0.85	1.23	1.07	0.47	0.66	0.94
19	1.16	0.75	1.08	0.90	1.01	0.57	0.83	0.79	1.25	0.85	1.25	0.85
20	0.70	0.16	0.42	0.58	0.32	0.26	0.39	0.25	0.94	0.37	0.65	0.75
21	0.93	0.19	0.67	0.58	1.74	0.49	0.72	1.53	1.26	0.61	1.00	0.95
22	0.49	0.28	0.53	0.36	1.70	0.87	0.96	1.60	0.81	0.58	0.78	0.61
23	0.53	0.32	0.21	0.68	0.24	0.53	0.29	0.51	0.53	0.48	0.35	0.78
24	2.07	0.41	1.79	0.77	1.82	0.69	1.86	0.89	2.19	0.68	2.05	1.05
25	1.01	0.39	0.68	0.89	2.38	1.14	1.26	2.15	0.59	0.60	0.73	0.51
26	1.22	0.37	0.66	1.25	0.80	0.44	0.76	0.51	2.38	0.73	1.00	2.31
27	1.03	0.34	0.62	0.85	0.99	0.36	0.74	0.56	2.29	0.52	0.89	1.79
28	1.73	0.54	1.53	0.75	1.66	0.55	1.57	0.76	1.74	0.67	1.61	0.82
29	0.98	0.47	0.98	0.54	4.12	1.46	1.80	3.63	1.38	0.72	1.10	1.11
30	1.00	0.41	0.95	0.49	1.26	0.55	1.11	0.67	1.27	0.61	1.17	0.78
31	1.48	0.59	0.83	1.49	2.54	1.24	1.37	2.66	1.48	0.89	1.00	1.45
32	1.14	0.40	0.54	1.24	2.28	0.93	0.93	2.40	1.20	0.57	0.59	1.22
average	1.04	0.44	0.83	0.73	1.50	0.73	1.05	1.22	1.34	0.67	1.02	1.03

**Table 5:** Evaluation of the UERE under different scenes (Shantou, Harbin and Urumchi, unit: m).

PRN	Shantou				Harbin				Urumchi			
	Scene1	Scene2	Scene3	Scene4	Scene1	Scene2	Scene3	Scene4	Scene1	Scene2	Scene3	Scene4
1	2.15	1.16	1.22	2.06	0.91	0.64	0.66	0.90	1.11	0.62	0.74	0.82
2	1.23	0.57	0.74	1.04	1.10	0.66	0.70	1.06	0.92	0.71	0.81	0.90
3	1.92	1.10	1.28	1.90	0.70	0.42	0.65	0.47	1.00	0.46	0.69	0.67
4	2.16	0.89	0.92	2.12	0.28	0.26	0.44	0.16	1.03	0.29	0.46	0.91
5	1.40	0.88	1.29	0.95	1.18	0.46	0.94	0.74	1.09	0.22	0.84	0.49
6	1.17	1.01	1.03	1.19	0.64	0.57	0.53	0.79	0.99	0.58	0.68	0.91
7	2.09	0.83	1.42	1.98	1.18	0.37	1.15	0.52	1.75	0.49	1.35	0.95
8	2.86	1.41	2.41	1.85	1.77	0.32	1.55	0.64	1.68	0.34	1.53	0.94
9	2.06	1.13	1.22	2.07	0.05	0.32	0.25	0.12	0.70	0.47	0.38	0.75
10	2.28	1.45	1.69	2.22	1.04	0.27	0.58	0.77	0.66	0.47	0.51	0.64
11	1.73	0.75	1.11	1.33	1.32	0.50	1.04	0.83	1.75	0.59	1.26	1.25
12	2.73	1.45	1.59	2.64	0.81	0.56	0.75	0.81	0.78	0.53	0.64	0.67
13	1.31	0.88	0.93	1.28	0.86	0.44	0.72	0.83	0.54	0.53	0.55	0.55
14	3.38	1.90	2.85	2.52	1.20	0.61	1.18	0.42	1.15	0.76	1.10	0.56
15	1.43	0.75	0.83	1.41	0.58	0.22	0.41	0.46	0.79	0.27	0.51	0.57
16	2.40	1.19	1.87	1.84	0.94	0.32	0.62	0.71	1.28	0.36	0.74	1.07
17	1.99	1.10	1.82	1.47	2.08	0.88	1.75	1.22	2.03	1.02	1.64	1.18
18	1.77	1.20	1.15	1.81	0.90	0.51	0.71	0.74	0.96	0.49	0.65	0.82
19	1.17	0.60	0.91	0.89	1.16	0.64	0.89	1.00	1.43	0.78	1.05	1.04
20	1.74	1.08	1.13	1.74	0.65	0.10	0.37	0.48	0.73	0.26	0.49	0.42
21	1.65	0.70	0.81	1.57	1.01	0.22	0.66	0.62	1.20	0.39	0.74	0.94
22	1.82	1.09	1.07	1.75	0.55	0.34	0.50	0.38	1.03	0.48	0.78	0.84
23	1.80	0.86	1.07	1.55	0.64	0.35	0.29	0.72	0.31	0.41	0.11	0.52
24	2.99	1.06	2.38	1.87	2.12	0.50	1.80	0.74	2.00	0.56	1.98	0.65
25	2.6	1.19	1.78	2.32	1.36	0.35	0.64	1.18	0.17	0.47	0.48	0.13
26	2.31	1.11	1.66	1.73	1.21	0.37	0.69	1.12	0.99	0.85	1.11	0.81
27	2.05	1.06	1.25	2.00	1.03	0.40	0.59	0.92	0.62	0.39	0.62	0.53
28	1.91	0.65	1.52	1.11	1.62	0.49	1.47	0.67	1.62	0.92	1.59	0.89
29	3.64	1.60	1.93	3.20	1.36	0.59	0.96	0.93	1.20	0.67	1.04	0.79
30	1.45	0.78	1.20	1.35	0.89	0.46	0.95	0.40	1.21	0.45	0.98	0.69
31	2.66	1.32	1.43	2.73	1.41	0.35	0.62	1.32	1.04	0.49	0.60	0.97
32	2.53	1.11	1.23	2.55	1.29	0.58	0.51	1.37	0.72	0.47	0.52	0.60
average	2.07	1.06	1.40	1.81	1.06	0.44	0.80	0.75	1.08	0.52	0.85	0.76

It could be seen that:

1. Compared with the accuracy of the GPS SPS UERE in the specified area, BDSBAS could effectively improve the accuracy when all or part of the correction parameters applied.
2. The accuracy could not be obviously improved with only the position and clock corrections or the ionospheric corrections applied.
3. As the satellite came in and out the service boundary, the variation range of the $UERE_{{{\text{SBAS}}}}$ is more gentle than the $UERE_{{{\text{SPS}}}}$. The variation scope of the scene 1 and scene 3 was quiet similar. It could be seen that the two kind of ionospheric correction mode did not have significant difference.

## Evaluation of the SF PE with/without the support of the BDSBAS-B1C message

**Figure 9:**
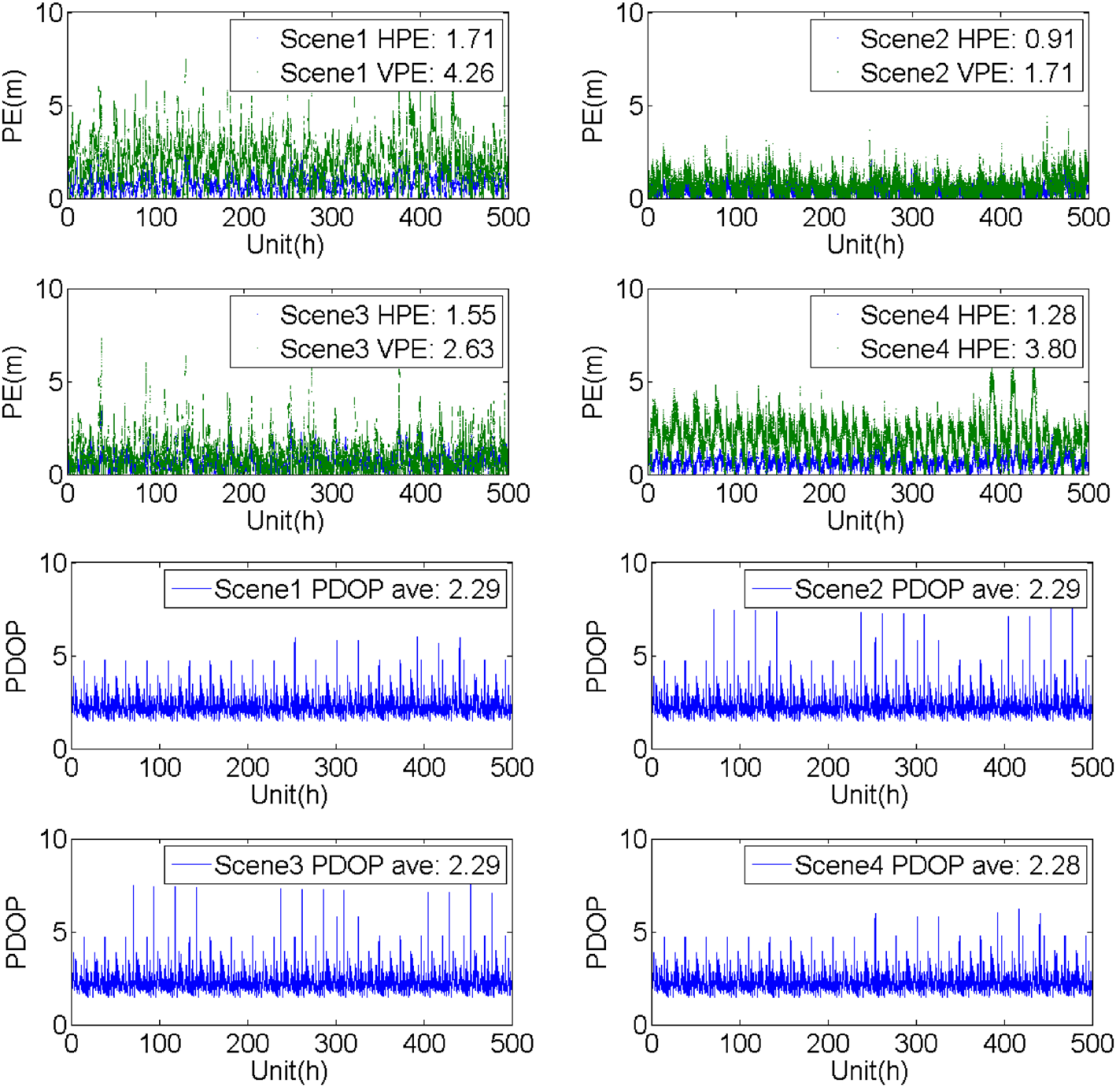
Evaluation of the PE and PDOP under different scenes in Beijing from Dec 11 to 31, 2020.

**Figure 10:**
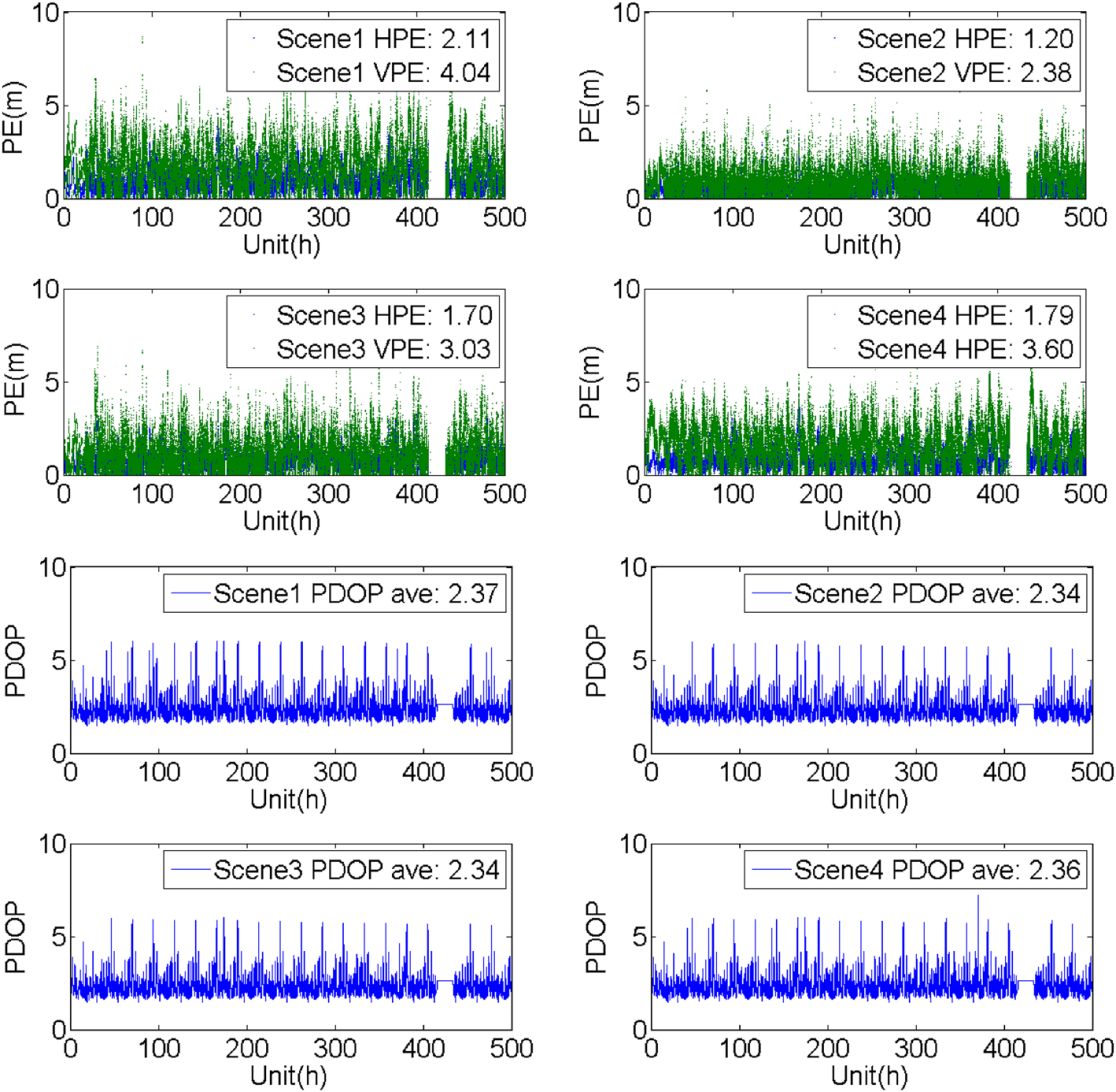
Evaluation of the PE and PDOP under different scenes in Chengdu known point from Dec 11 to 31, 2020.

**Figure 11:**
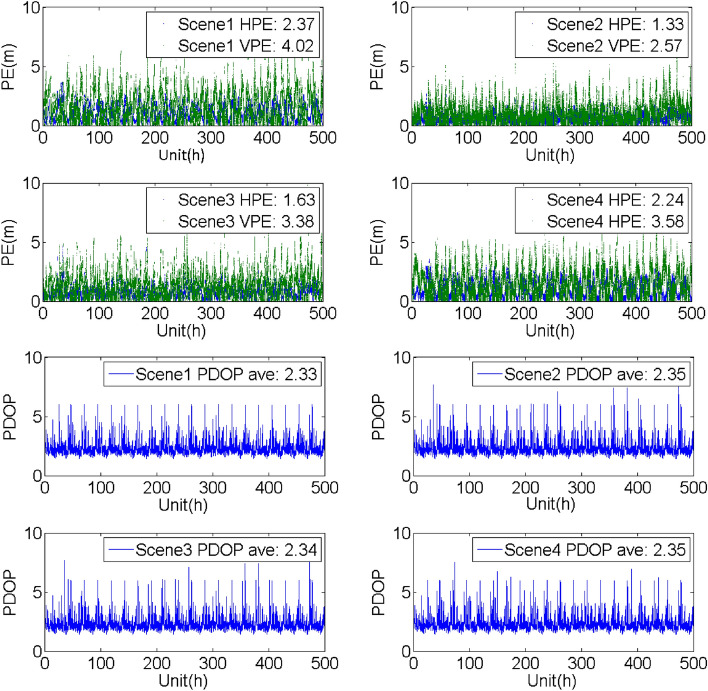
Evaluation of the PE and PDOP under different scenes in Sanya known point from Dec 11 to 31, 2020.

**Figure 12:**
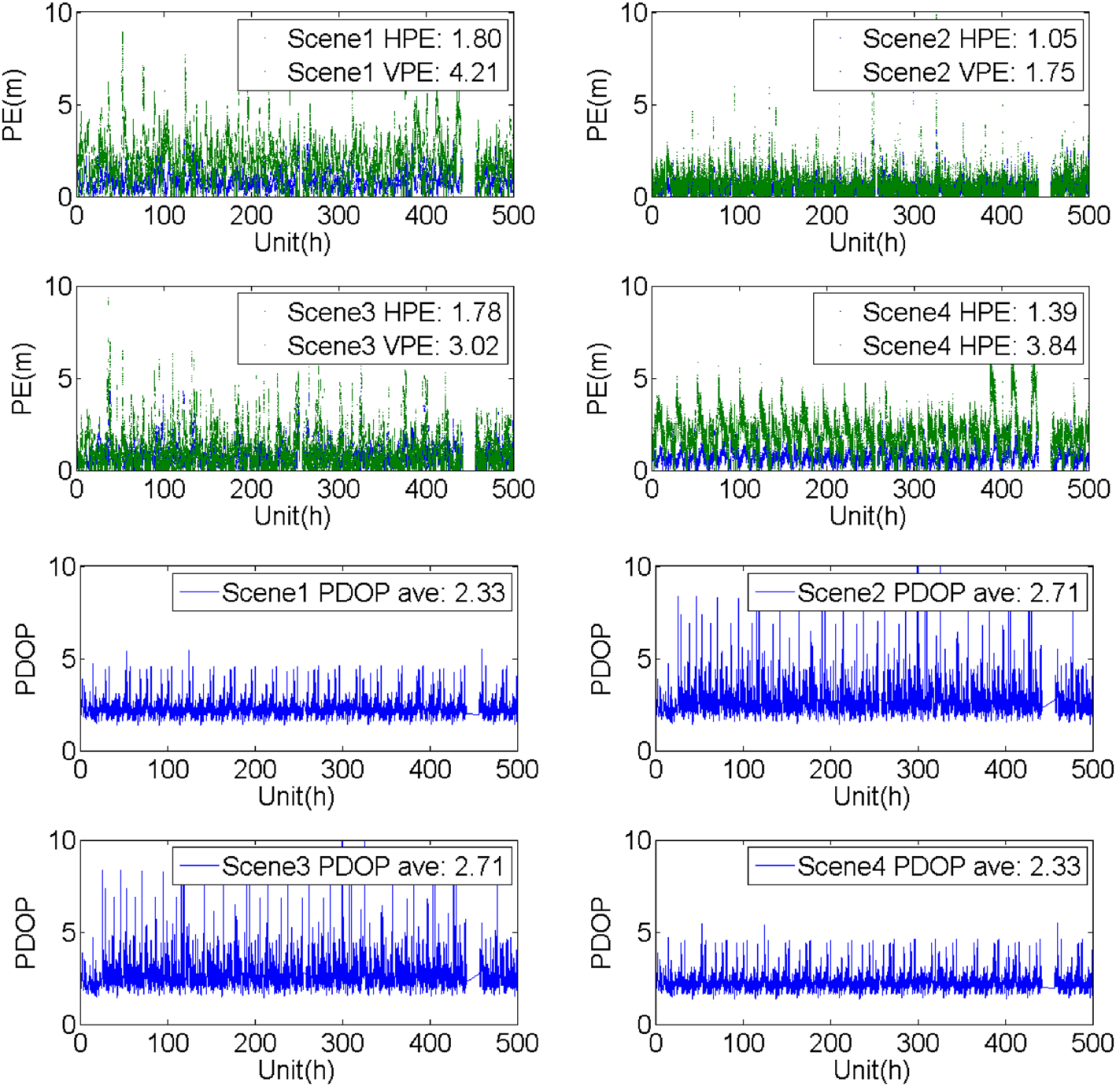
Evaluation of the PE and PDOP under different scenes in Harbin known point from Dec 11 to 31, 2020.

To analyze the SF PE of the GPS satellites with BDSBAS support, four groups of experiments were carried out with the observation data and navigation message obtained from the known points in different service areas from Dec 11 to 31, 2020. The four constructed scenes were the same as the scenes mentioned above. The horizontal positioning error (HPE), vertical positioning error (VPE) and the corresponding PDOP were given in Table 6, Figs. 9, 10, 11 and 12.

**Table 6:** Evaluation of the SF PE under different scenes (95%, PDOP < 6, unit: m).

Known point	Scene1		Scene2		Scene3		Scene4	
	HPE	VPE	HPE	VPE	HPE	VPE	HPE	VPE
Beijing	1.71	4.26	0.91	1.71	1.55	2.63	1.28	3.80
Sanya	2.37	4.02	1.33	2.57	1.63	3.38	2.24	3.58
Chengdu	2.11	4.04	1.20	2.38	1.70	3.03	1.79	3.60
Haerbin	1.80	4.21	1.05	1.75	1.78	3.02	1.39	3.84
Urumchi	1.98	3.87	1.54	2.46	3.81	7.57	1.47	3.42
Kuerle	1.83	4.13	0.94	1.44	1.58	2.31	1.37	3.68
Baotou	2.00	4.27	1.39	2.17	1.82	2.86	1.70	3.99
Jiamusi	1.80	4.46	1.43	2.56	2.82	5.05	1.41	4.09
Jinghong	3.05	4.59	1.80	2.89	2.16	3.86	2.91	3.86
Guiyang	2.90	3.93	1.54	2.36	1.92	3.11	2.69	3.36
Xiangyang	2.01	4.45	1.31	2.90	1.78	3.45	1.65	4.05
Zhoushan	2.21	3.94	1.89	3.55	3.07	5.73	1.90	3.61
Dunhuang	1.82	5.33	0.94	2.67	1.63	3.35	1.30	4.78
Average	2.12	4.27	1.33	2.42	2.10	3.80	1.78	3.82

It could be seen that:

1. Compared with the single point PE of the GPS SPS obtained by the navigation message broadcasted by the GPS satellites, BDSBAS could effectively improve the positioning accuracy with all the correction parameters applied. Whereas, the accuracy could be hardly improved when only the position and clock corrections or the ionospheric corrections were applied.
2. Through the comparison of the different scenes, it could be seen that the positioning accuracy in the vertical direction has more improvements than the accuracy in the horizontal direction. It may benefit from the effective correction of the ionosphere in the vertical direction.
3. The PDOP indicated the availability of the correction parameters for the visible satellite. For the regions with high availability of the ionospheric corrections, such as Beijing and Chengdu, the PDOP was almost the same in different scenes and the PE was mainly affected by the accuracy of the correction parameters. For the boundary area of the BDSBAS service, such as Harbin, there was obvious difference in different scenes. The reason for the lower PDOP in scene2 and scene3 was probably that the ionospheric corrections were applied in scene2 and scene3 and the availability of the ionospheric corrections was low.

## Summary

Currently, BDSBAS has realized the broadcasting of the augmentation message with three GEO satellites and constructed along with other BDS-3 services. The conditions for initial operation have been met. Though the above analysis, the basic conclusion could be gain as follows:

1. The construction and content of the augmentation message broadcasted in the BDSBAS-B1C signal has basically met the requirement of the RTCA DO-229E. Whereas, the offset parameters of BDSBAS SNT and UTC and the SBAS service message have not been broadcasted.
2. BDSBAS has specified a broadcasting strategy of the BDSBAS-B1C message with reference to the standards published by the ICAO, RTCA and other international organizations. Whereas, the fixed timing sequence may be more suitable for the case of no alarm events as the message containing the alert condition should be broadcasted four times in four seconds and relace the fixed message. It should be further verified if it is suitable in the case of alarm.
3. In contrast to the accuracy of the UERE and single point PE obtained by the navigation message of the GPS satellites in the specified area, the BDSBAS could effectively improve the monitoring results. The results showed that the accuracy and availability of the ionospheric parameters was one of the most important factors. To satisfy the requirements of Category I precision approach (CAT-I) as WAAS and EGNOS, more effort should be put into to improve the availability of the ionospheric corrections in the boundary areas.

## Data Availability

The data was provided by the BDSBAS civil service platform and the international GNSS Monitoring and Assessment System (iGMAS).
